# *Aeromonas allosaccharophila* Strain AE59-TE2 Is Highly Antagonistic towards Multidrug-Resistant Human Pathogens, What Does Its Genome Tell Us?

**DOI:** 10.3390/life12101492

**Published:** 2022-09-26

**Authors:** Sheila da Silva, Fernanda Alves de Freitas Guedes, João Ricardo Vidal Amaral, José Roberto de Assis Ribeiro, Yuri Pinheiro Alves de Souza, Ângela Correa de Freitas-Almeida, Fabiano Lopes Thompson, Rommel Thiago Jucá Ramos, Andrew Steven Whiteley, Andrew Macrae, Selma Soares de Oliveira

**Affiliations:** 1 Programa Pós-Graduação de Biotecnologia Vegetal e Bioprocessos da Universidade Federal do Rio de Janeiro, Av. Prof. Rodolpho Paulo Rocco, s/n-Prédio do CCS-Bloco K, 2° Andar-Sala 032, Rio de Janeiro 21941-902, Brazil; 2 Helmholtz Zentrum Munchen COMI, Research Unit Comparative Microbiome Analysis, 85764 Neuherberg, Germany; 3 Departamento de Microbiologia, Imunologia e Parasitologia, Centro Biomédico, Faculdade de Ciências Médicas, Universidade do Estado do Rio de Janeiro (UERJ), Av. 28 de Setembro, 87, 3° Andar, Fundos, Vila Isabel, Rio de Janeiro 20550-170, Brazil; 4 Departamento de Genética, Instituto de Biologia, Universidade Federal do Rio de Janeiro, Av. Prof. Rodolpho Paulo Rocco, s/n-Prédio do CCS-Instituto de Biologia, 2° Andar-Sala 93, Rio de Janeiro 219410-970, Brazil; 5 Instituto de Ciências Biológicas, Centro de Genômica e Biologia de Sistemas da Universidade Federal do Pará (UFPA), Rua Augusto Corrêa, 01 Guamá, Belém 66075-970, Brazil; 6 Commonwealth Scientific and Industrial Research Organization (CSIRO), Canberra, ACT 2601, Australia; 7 Instituto de Microbiologia Paulo de Góes da Universidade Federal do Rio de Janeiro, Av. Prof. Rodolpho Paulo Rocco, s/n-Prédio do CCS-Bloco I, 1° Andar-Sala 047, Rio de Janeiro 21941-902, Brazil

**Keywords:** *Aeromonas allosaccharophila*, antimicrobial activity, bacteriocins, antimicrobial resistance, genomics

## Abstract

Multidrug-resistant bacteria are of critical importance and a problem for human health and food preservation; the discovery of new antimicrobial substances to control their proliferation is part of the solution. This work reports on 57 antagonistic *Aeromonas* strains, of which 38 strains were antagonistic towards problematic human pathogens. The genome of the most antagonistic strain was sequenced and identified as *Aeromonas allosaccharophila*. Its genome was fully annotated and mined for genes that might explain that activity. Strain AE59-TE was antagonistic toward clinically relevant gram-negative and gram-positive multidrug-resistant bacteria, including *Klebsiella pneumoniae* KPC, *Escherichia coli* ESBL, *Salmonella typhimurium*, and *Staphylococcus aureus* MRSA. Strain AE59-TE2 was identified by multilocus sequence analysis. Genome mining identified four genes homologous to the bacteriocin, zoocin A from *Streptococcus equi* and a gene 98% similar to *cvp*A linked to colicin V production. *A. allosaccharophila* strain AE59-TE2 produced antimicrobial activity against a broad range of bacteria, including important gram-negative bacteria, not typically targeted by bacteriocins. Herewere described novel zoocin genes that are promising for industrial applications in the food and health sectors. Interesting and important antagonistic activity is described combined with the first detailed genomic analysis of the species *Aeromonas allosaccharophila*.

## 1. Introduction

Multidrug-resistant bacteria cause persistent hospital infections that increase morbidity and mortality, especially in developing countries [1,2]. Their impact on health care systems is mostly due to the unavailability of effective antibiotics [1]. The main nosocomial antibiotic-resistant pathogens are *Acinetobacter baumannii*, *Pseudomonas aeruginosa*, extended-spectrum beta-lactamase-producing *Escherichia coli*, methicillin-resistant *Staphylococcus aureus* (MRSA), *Klebsiella pneumoniae*, carbapenem-resistant *Enterobacterales* (CRE), and vancomycin-resistant *Enterococci* (VRE) [1,3,4]. Antimicrobial resistance is the ability of microorganisms to inactivate or decrease the effectiveness of antibiotics. Resistance can occur spontaneously due to genetic modifications; nonetheless, this process can be accelerated by the inappropriate use of antibiotics, resulting in evolutionary pressures for genetic mutations and the exchange of genetic material between bacteria and phages [5]. Since the discovery of antibiotics, between 1930–1962, more than 20 new classes have been described. However, resistance continues to evolve, and the search for new antimicrobial compounds is an urgent challenge [5]. Only three new classes of antibiotics against gram-positive bacteria have been described recently: the Oxazolidinones class with Linezolid (2001) and tedizolid (2014); the daptomycin class, consisting of cyclic lipopeptides, discovered in 2006; and the fidaxomicin class, a macrocycle drug, discovered in 2011 [5].

Bacteria are a source of many antimicrobial compounds. They produce lipopeptide, comprising non-ribosomal peptides synthetases (NRPSs), such as circular lipopeptides (surfactin, iturine, and phengycine families), polyketide (PKS) compounds, and siderophores [6]. Some of these compounds are products of secondary metabolism, such as antibiotics, while others are bioactive molecules ribosomally synthesized, such as antimicrobial peptides and bacteriocins. Bacteriocins are viable alternatives to antibiotics that are no longer effective due to antimicrobial resistance [7,8]. Traditional bioprospecting strategies for new antibiotics are not efficient in finding new substances [9]. Since traditional methods for screening antimicrobial substances can last a long time and have high costs, genomic analyses provide a new opportunity to search these substances in a more practical and less expensive way. Genome sequencing, gene annotation, and the activation of silent gene clusters constitute the basis of new methods for massive screening and new antibiotics discovery and yet success is limited [9].

*Aeromonas* strains are known to produce several antimicrobial substances with the potential to become new antibiotics and therefore are worthy of detailed genomic investigations. The *Aeromonas* genus comprises gram-negative, facultative anaerobic bacteria often found in aquatic environments [10], the human gastrointestinal tract, and other animals, including fish, reptiles, and amphibians [10,11,12]. *Aeromonas* species can cause several animal diseases. Furunculosis, for example, is a condition observed in fish [13], which is associated with significant economic losses in pisciculture [11,12,14]. Many *Aeromonas’* virulence genes have already been reported: *vapA* (layer A); *act*, *alt*, and *ast* (cytotonic enterotoxins); *ahyB* (elastase); *exu* (DNases) [15,16]. *Aeromonas* strains are considered opportunistic pathogens, infecting mainly immunosuppressed patients [11,12,17]. Although there are reports correlating *Aeromonas* sp. to gastroenteritis and few cases of more severe infections, the etiological role of the genus in this pathogenicity remains controversial [17,18]. The World Health Organization’s “One-World-One Health” concept highlights that healthiness is based on a balance of human, animal, microbe and environmental interactions [19]. In this manner, solutions to these problems are likely to be found in nature. Antagonistic interactions are continually observed and are a part of nature, and are in natural environments. This concept was a guide for the research presented in this article. Bacteriocins receive special focus because they possess great potential in preventing the spread of infectious bacteria, controlling spoilage of industrialized products, and mitigating the indiscriminate and excessive use of other antibiotics [20].

There are numerous reports on *Aeromonas* strains producing bacteriocin-like substances (BLS) [21,22]. However, to date, the activity and presence of BLS has not been linked to its genetic origin. Antimicrobial peptides are important compounds for microorganisms, which grant competitiveness in different environments [23]. These molecules are synthesized by several organisms for their defense. Amongst these are peptides called bacteriocins, which can kill or inhibit the growth of other microorganisms [7]. Bacteriocins from gram-positive bacteria are frequently described as inhibiting other gram-positive strains [24]. However, important gram-negative pathogens, such as *Salmonella* and *Escherichia coli*, have not yet been targeted by bacteriocins [25,26]. Thus, there is a need to discover and report on bacteriocins that target gram-negative disease-causing bacteria.

Biotechnological applications of bacteriocins include their use as antibiotics, food preservatives and bacteriocin, such as Nisin, used by food industries [8,23]; and as probiotics [27]. Bacteriocins may also have applications as anticancer agents [7,24]. There are new assays that use bacteriocins in agriculture for the biocontrol of phytopathogens [8]. Colicin is used for the biocontrol of pests in tobacco plants and considered an efficient strategy that meets GRAS (FDA) safety protocols for controlling bacteria [28]. Bacteria from aquatic environments have already been described as great candidates for the production of antimicrobial substances. The *Aeromonas* genus has been reported as capable of producing bacteriocins. This is an interesting genus, since it is found either in animal, water, or human, which requires a certain level of adaptation to different niches, where bacteriocins and toxins may play a role in the competition and maintenance of those species in respective niches [29]. Therefore, our work aimed to isolate *Aeromonas* bacteria from fish to investigate the antimicrobial substances’ production and to perform the genomic characterization of the producing strain. Here are described the bioprospecting and screening of a wide range of wild *Aeromonas* strains looking for novel antagonistic behavior, followed by genomic mining to search for genes related to bacteriocins and antimicrobial activity.

## 2. Materials and Methods

### 2.1. Sample Collection and Isolation

*Aeromonas* strains were isolated from healthy fish branchiae, scales, and cloaca. Two replicates were taken for *Mugil brasiliensis* (popular names: Tainha/Mullet) and three replicates for *Caranx latus* (popular name: Xerelete), which were purchased at a street market located in Rio de Janeiro city, RJ, Brazil (−22.910468, −43.240857). A total of 200 mL of water was collected from six different lagoon points at the Rodrigo de Freitas Lagoon (latitudes 43°11′09″ N and 43°13′03″ S, and longitudes 022°57′02″ E and 022°58′09″ W). Water samples were centrifuged at 12,100× g for 15 min and the pellet was used to isolate *Aeromonas*. One *Aeromonas* strain previously isolated from lettuce leaves [30] was also screened for antagonistic activity in this bioprospection. All the samples collected were incubated in alkaline peptone water (APA) at 30 °C for 24 h and were inoculated at 30 °C for 48 h in selective medium glutamate starch phenol (GSP) red agar (Merck, Darmstadt, Germany). The *Aeromonas* sp. strains were then examined and classified into phenospecies using the criteria described in the literature [12,31].

### 2.2. Antimicrobial Activity Screening

A total of 57 *Aeromonas* strains were screened for antimicrobial activity by either the agar well diffusion assay [32] or the chloroform method [33] with modifications (Supplementary Figure S1). Both experiments were performed in triplicate. Bacteria were grown on nutrient agar and were incubated at 28 °C. Inhibition halos greater than 1 cm were considered as positive results for antimicrobial activity. *Klebsiella pneumoniae* KPC (*Klebsiella pneumoniae* carbapenemase), *K. pneumoniae* ESBL (extended-spectrum β-lactamase-producing), *K. pneumoniae* ATCC 13883, *Escherichia coli* ESBL, *Enterobacter cloacae* NDM (New Delhi metallo-beta-lactamase), *Acinetobacter baumannii*, *Salmonella typhimurium* ATCC 14028, *Pseudomonas aeruginosa*, and *P. aeruginosa* strains SPM (São Paulo metallo-β-lactamase) from Laboratory of Medical Investigation; *E. coli*, *Staphylococcus aureus* ATCC 6538 and *P. aeruginosa* ATCC 15422 from Laboratory of Food Microbiology and *K. pneumoniae* 19ae, *Enterococcus faecalis* 5ae, *S. aureus* HIV 86a, and *S. aureus* HIV 87a from the Laboratory of Nosocomial Infection were used as indicator strains. These strains were selected because they can be etiological agents of severe diseases and are multidrug resistant. The presence (value 1) or absence (value 0) of antimicrobial activity was converted into a table and used to build a hierarchical clustering with the GenePattern online tool [34] using its default values. Strain AE59-TE2 exhibited the broadest antimicrobial activity spectrum and was selected for genome sequencing and mining.

### 2.3. DNA Extraction, Illumina Sequencing, Data Preprocessing, and Genome Assembly

Genomic DNA was extracted and purified using the CTAB method [35]. The AE59-TE2 paired-end (2 × 300 bp) library was constructed from approximately 1 µg of gDNA using the Nextera XT DNA Sample Preparation Kit (Illumina, San Diego, CA, USA) and sequenced with the MiSeq Illumina platform (Rio de Janeiro, Brazil). Trimmomatic v0.36 [36] was used for quality control and to trim the sequences using default parameter values to remove adaptors and N-containing reads, as well as small (<36 bp) reads. SPAdes v3.10.1 [37] was used for a de novo assembly, and contigs were mapped twice with the MeDuSa v.1.6 server [38] using 6 *A. veronii* complete genomes (GCA_001634325.1, GCA_001593245.1, GCA_001634345.1, GCA_002803925.1, GCA_002803945.1, and GCA_000204115.1) as reference, being the closest species and with the most complete genomes deposited on NCBI. To evaluate the assembly’s quality, filtered reads were aligned to the AE59-TE2 scaffolds using BWA v0.7.75a [39] and the statistics were obtained with Qualimap v2.2.1 [40]. CheckM v1.4.0 [41] was used to verify genome completeness and contamination, and QUAST v.5.0.2 [42] was used to verify the genome quality. A flowchart summarizing the assembly steps is shown in Supplementary Figure S2. The AE59-TE2 sequenced library and the genome final assembly were deposited at the NCBI database under BioSample accession numbers SAMN08436981.

### 2.4. Taxonomic Identification by Molecular Methods

Multiple methods were used for species identification: *16S rRNA* gene sequencing and phylogenetic analysis with BLAST server v.2.12.0 [43], multilocus sequence analysis (MLSA), in silico DDH using the Genome-to-Genome Distance Calculator (GGDC) v2.1 server [44] were performed. In silico DDH analyses, using 33 *Aeromonas* Type strains (Supplementary Table S1) obtained from the EZBio Cloud database (https://www.ezbiocloud.net/ accessed on 18 September 2022) were made with a cutoff value of 70% of similarity. MLSA was conducted with six housekeeping genes (*recA*, *gyrB*, *gltA*, *metG*, *groL*, and 16S rRNA) [45] from *A. allosaccharophila* CECT 4199, *A. aquatica* AE235, *A. australiensis* CECT 8023, *A. bestiarum* CECT 424227, *A. bivalvium* CECT 7113, *A. caviae* CECT 838, *A. dhakensis* CIP 1077500, *A. diversa* CECT 4254, *A. encheleia* CECT 4342, *A. enteropelogenes* CECT 4487, *A. eucrenophila* CECT 4224, *A. finlandensis* 4287, *A. fluvialis* LMG 24681, *A. hydrophila* subsp. *hydrophila* ATCC 7966, *A. jandaei* CECT 4228, *A. lacus* AE122, *A. media* CECT 4232, *A. piscicola* LMG 24783, *A. popoffii* CIP 105493, *A. rivuli* DSM 22539, *A. salmonicida* subsp. *masoucida* NBRC 13784, *A. salmonicida* subsp. *pectinolytica* 34mel, *A. sanarellii* LMG 24682, *A. simiae* CIP 107798, *A. sobria* CECT 4245, *A. taiwanensis* LMG 24683, *A. tecta* CECT 7082, and *A. veronii* CECT 4257. *Oceanimonas doudoroffi* ATCC 27123 was used as an outgroup. All genomes were annotated with the Rapid Prokaryotic Genome Annotation (PROKKA) tool v1.14.6 [46]. For the multilocus sequence analysis, sequences of each gene were concatenated to construct “supergenes” (approximately 14 kbp). They were then multiple aligned and gaps were removed using BioEdit v7.2.5 [47]. Phylogenetic analyses were conducted using MEGA11 software [48]. A phylogenetic tree was inferred using the Maximum Likelihood (ML) method and General Time Reversible (GTR) model. A discrete Gamma distribution was used to model evolutionary rate differences among sites, allowing for some sites to be evolutionarily invariable. The bootstrap test was performed using 1000 replicates. The taxonomic classification was corroborated by the Average Nucleotide Identity (ANI) v3.8.3 and Tetra Correlation Search (TCS) v3.8.3 analyses using the AE59-TE2 genome against GenomesDB in JSpeciesWS server v3.8.3 [49]. ANIb result was shown by heatmap using R tool [50].

### 2.5. Virulence Potential and Antibiotic Resistance

The search for virulence factors is important, as there are already reports of bacteriocins with typical characteristics of virulence factors, thus making dissemination and replication in the host cell easier [29,51]. The virulence potential of the AE59-TE2 strain was evaluated using the virulence factors database (VFDB) from the ABRicate tool version 1.0.1 [52]. NCBI Antimicrobial Resistance Gene Finder Plus (AMRFinderPlus) tool v3.10.23 [53], ResFinder v 4.0 [54], and Resistance Gene Identifier (RGI) v.5.2.0 from Comprehensive Antibiotic Resistance Database (CARD) v. 3.1.4 [55] were used to verify the antibiotic resistance profile.

### 2.6. Genome Functional Annotation and Mining

Gene prediction and functional annotation were carried out using the classic RAST v2.0 server [56] and Rapid Prokaryotic Genome Annotation (PROKKA) tool v1.14.6 [46]. To search for more genes related to bacteriocin production, BAGEL (class III), Bactibase, and DoBiscuit-Database of BioSynthesis cluster CUrated and InTegrated (https://www.nite.go.jp/nbrc/pks/ accessed on 18 September 2022) [57] and some genes for the Colicin V production protein (*cvaC*) and Zoocin production protein (ZooA) from UniProt were used with BLASTp v.2.11.0+ [42] against the PROKKA genome annotation. Four zoocin-like sequences from the AE59-TE2 genome and 1 zoocin A sequences from UniProt (accession number: O54308) was multiple aligned with Clustal Omega v1.2.4 [58]. Analysis with AntiSMASH v6.0 was performed to verify secondary metabolism and search for bacteriocins [59]. The GO FEAT tool [60] was used for functional annotation and enrichment of genomic data. The KEGG Automatic Annotation Server (Kaas) [61] and KEGG Mapper Reconstruction were used to make orthology assignments and pathway mapping. To perform a better characterization of the genome, the Pathosystems Resource Integration Center (PATRIC) v.3.6.9 was used [62], and the GO Feat tool was used to search for keywords that are related to antimicrobial activities, such as the words: Bacteriocin, Antibiotic, Colicin, Microcin, Endopeptidase, Endonuclease, polyketides (PKS), and Rhamnolipid. The circular genome plot was made with the Circular Genome Viewing (CGView) tool [63].

## 3. Results

### 3.1. Sample Identification and Phenotypic Characterization of Antibacterial Activity of Aeromonas Isolates

**Figure 1:**
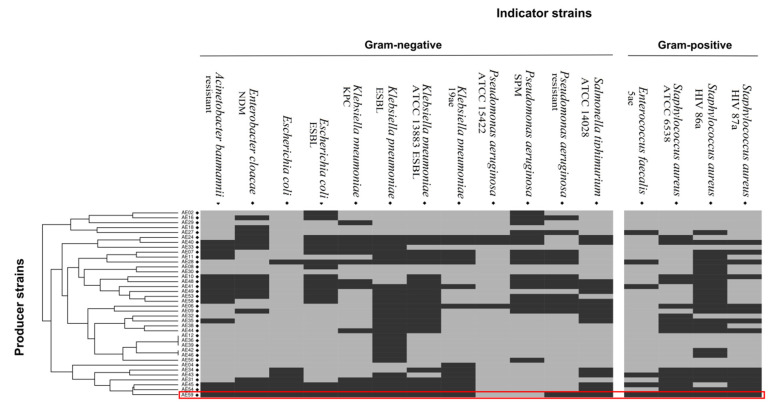
Antimicrobial activity tests using *Aeromonas* sp. producer strains against clinical and potentially pathogenic indicator strains. The results of the antimicrobial activity tests were converted into positive (1) and negative (0) antimicrobial activity. A table of this data was used as an input for a hierarchical clustering. The numerical values were then converted into a color-code, where dark grey indicates that an inhibitory activity was observed.

Forty-one *Aeromonas* strains were isolated from fish samples. Preliminary biochemical tests identified the samples as *A. hydrophila* (n = 21), *A. caviae* (n = 14), and *A. veronii* bv *sobria* (n = 6). Fifteen *Aeromonas* strains were isolated from the water sample and identified as *A. caviae* (n = 8), *A. hydrophila* (n = 4), *A. veronii* bv *sobria* (n = 2), and *A. salmonicida* (n = 1). Another *Aeromonas* strain previously isolated from lettuce was identified as *A. caviae* [30]. Among the 57 strains tested, 38 demonstrated differing levels of antimicrobial activity towards at least one highly pathogenic bacterial strain (Figure 1). A hierarchical cluster analysis was performed to visualize the results for antimicrobial activity. The analysis revealed a group of seven strains (AE04, AE34, AE43, AE31, AE45, AE54, and AE59-TE2) with a similar profile, broadly inhibiting both gram-negative and gram-positive multidrug-resistant pathogens. Strain AE59-TE2 exhibited antimicrobial activity towards 14 of the 16 indicator strains, namely *K. pneumoniae* (KPC, ESBL, 19ae ATCC 13883), *E. coli* and *E. coli* ESBL, *E. cloacae* NDM, *A. baumannii*, *S. typhimurium* ATCC 14028, *S. aureus* (ATCC 6538, HIV 86a, and HIV 87a), *E. faecalis* 5ae, and *P. aeruginosa* (Figure 1). Supplementary Figure S1 shows the inhibition zones produced against the *E. coli* ESBL strain.

### 3.2. AE59-TE2 Genome Sequencing and Assembly

**Figure 2:**
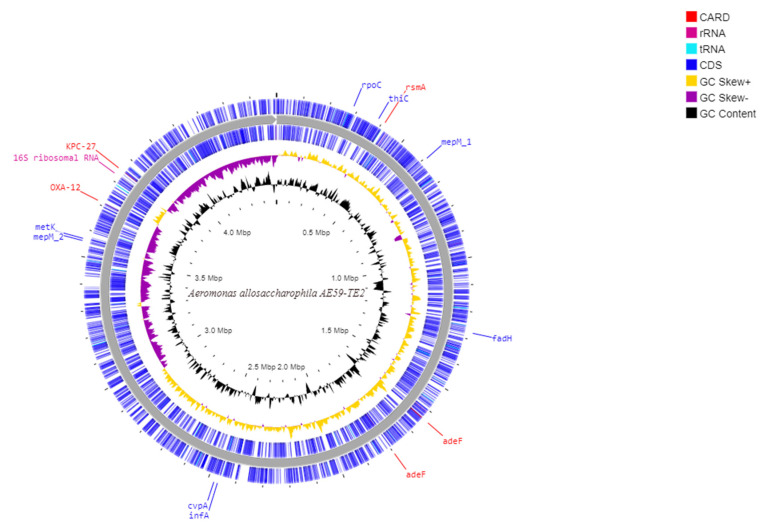
Schematic circular diagram of the AE59 genome highlighting the main sequences cited in the text. From the most external circle inwards: first and Second (blue)-CDS in positive strand; and negative strand; The purple circle is a GC-skew in negative strand and in yellow is a GC-skew in a positive strand; the black circle is a G + C content. The genes annotated with the CARD tool are marked with red; rRNA are marked with pink and the tRNA are marked with light blue.

Illumina MiSeq paired-end sequencing of the AE59-TE2 library yielded 532,201 raw reads. After preprocessing steps, 365,719 (68.72%) quality reads were obtained (Supplementary Table S2). De novo genome assembly generated 109 contigs (Table 1) and the mapping genome assembly resulted in one scaffold with a total sequence length of 4,498,261 bp and 58.68% G + C content in the QUAST result (Figure 2). CheckM analysis resulted in 100% completeness and 0.29% of contamination. More than 99% of the reads aligned to the assembled AE59-TE2 scaffolds, with a mean coverage of 23.42. Six AE59-TE2 scaffolds (scaffold_10, scaffold_20, scaffold_22, scaffold_26, scaffold_28, and scaffold_35) generated a consensus sequence (549 bp) that putatively encodes for a transposase (data not shown).

### 3.3. AE59-TE2 Species Identification and Aeromonas Taxonomy

**Figure 3:**
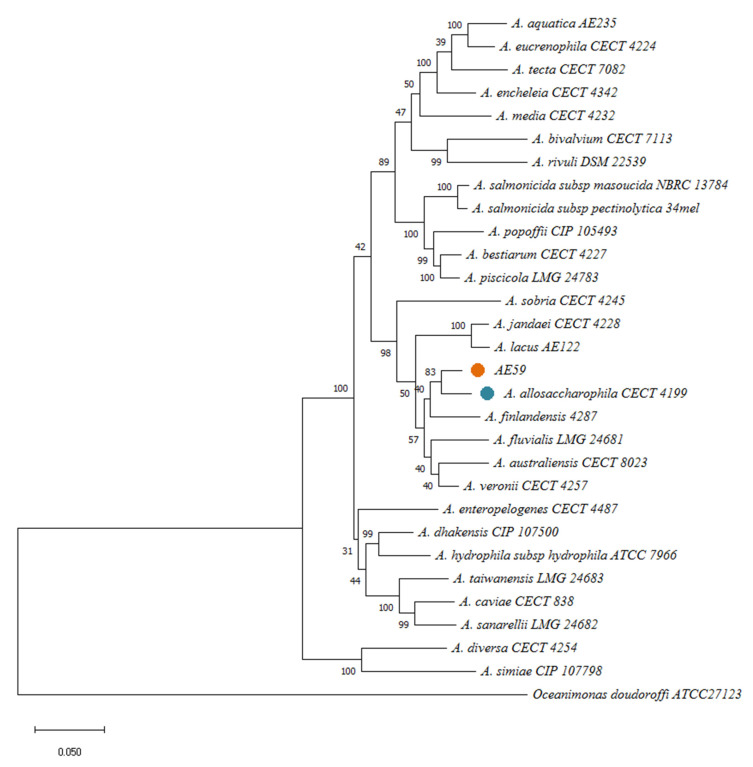
MLSA resultant phylogenetic tree, using six housekeeping genes: *recA*, *gyrB*, *gltA*, *metG*, *groL*, and *16S rRNA* and 28 genomes from *Aeromonas* genus, inferred with Maximum Likelihood (ML) method and General Time Reversible model. The model evolutionary used was a discrete Gamma distribution. The bootstrap consensus was inferred from 1000 replicates. *Oceanimonas doudoroffi* ATCC 27123 was used as an outgroup.

**Figure 4:**
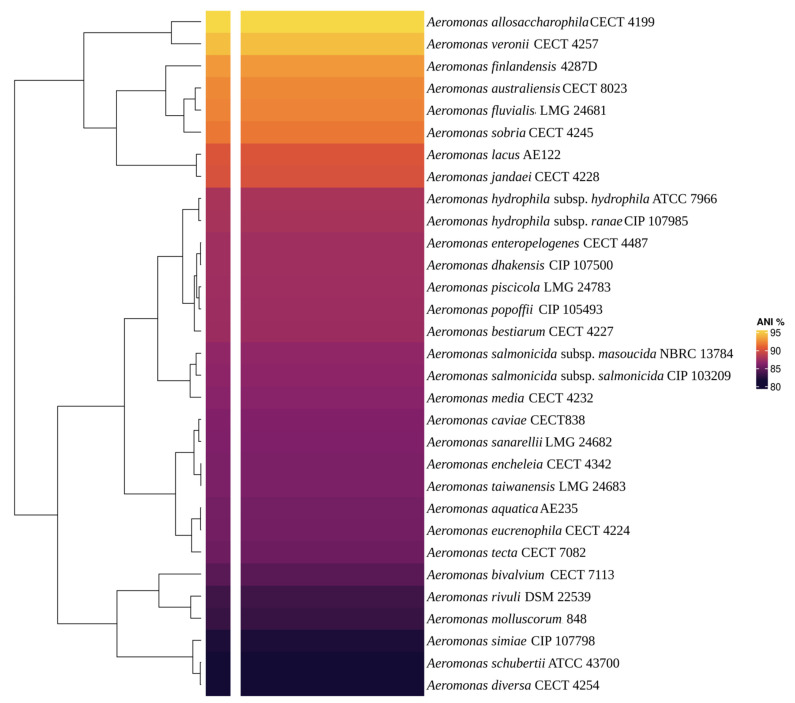
The heatmap showed the ANIb result. The main *Aeromonas* genus type strain was used to compare with the AE59-TE2 genome. The yellow color shows the most similar genome when compared with the AE59-TE2 genome. The value of yellow is 95.48% and represents *A. allosaccharophila* CECT 4199 genome.

Via BLAST and the nr database at the NCBI, the AE59-TE 16S rRNA sequence was highly similar to *Aeromonas allosaccharophila* (accession number: FJ940841.1; 100% identity and 99% query coverage) and *A. veronii* (accession number: CP024933.1; 99% identity and 100% query coverage). Since 16S rRNA analyses are not gold standard for species-level identification in the *Aeromonas* genus, MLSA, DDH in silico, and ANI analyses were performed. The MLSA phylogenetic tree inference grouped the AE59-TE2 strain and *A. allosaccharophila* together (Figure 3). DDH in silico analysis demonstrated that the *A. allosaccharophila* reference genome from EZBio Cloud database (Supplementary Table S3) was the most similar to the AE59-TE2 strain, DDH 62.6%. ANI analysis resulted in a score of 95.01% for type strain *A. allosaccharophila* CECT 4199 (Figure 4). TCS analysis resulted in a score of 0.99965 for *A. allosaccharophila* TTU2014-159ASC and a score of 0.99943 for type strain *A. allosaccharophila* CECT 4199. Analyses of the 16S rRNA gene, MLSA, DDH in silico, and ANI strongly indicate that the AE59-TE2 strain belongs to the *A. allosaccharophila* species cluster.

### 3.4. Virulence Potential and Antimicrobial Resistance

The virulence factors database (VFDB) identified 36 genes with identities above 90% and 88 genes with identities between 80–89% (Supplementary Table S4). Type III secretion system (T3SS) structural genes were identified (*ascV* and *ascC* genes), but only one gene of the main effectors was found (*aopH*). The ResFinder database did not identify any resistance genes in the AE59-TE2 genome. However, the AMRFinderPlus tool identified three genes: *blaOXA* (OXA-12 family class D beta-lactamase) with 98.11% identity and 100% coverage; *arsD* (arsenite efflux transporter metallochaperone) with 47.89% identify and 100% coverage; and *arsC* (glutaredoxin-dependent arsenate reductase) with 77.86% identity and 99.29% coverage. CARD/RGI annotated four strict hits. Two genes related to the resistance-nodulation-cell division (RND) antibiotic efflux pump: the *rsmA* gene with 92.73% identity; and *adeF* with 48.56% identity. A gene related to OXA beta-lactamase (antibiotic inactivation): the *OXA-726* with 95.45% identity. A gene related to elfamycin antibiotic (antibiotic target alteration), *Escherichia coli* gene EF-Tu mutants conferring resistance to Pulvomycin with 88.8% identity. This latter gene was annotated as a *tuf1* gene with 99.5% identity in the UniProt database and functions promoting the GTP-dependent binding of aminoacyl-tRNA to the A-site of ribosomes during protein biosynthesis.

### 3.5. Genome Mining of Aeromonas allosaccharophila AE59-TE2

**Figure 5:**
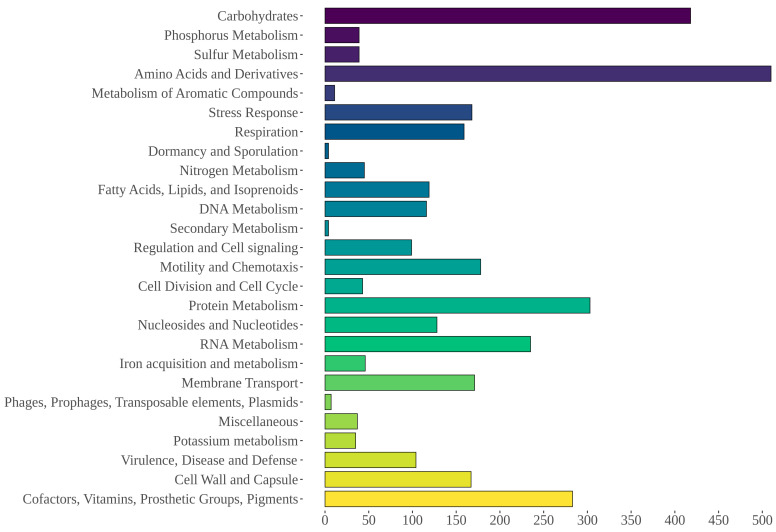
Functional RAST categories annotated for AE59 predicted sequences.

Gene annotation with the PROKKA software resulted in 4075 coding sequences, 10 rRNA, and 105 tRNA. The RAST server annotated 4173 features, including 4050 protein-coding sequences and 123 non-coding RNAs including tRNAs and rRNAs, with 2177 (52.17%) of them being categorized in at least one RAST-defined functional category (Figure 5). Five sequences associated with “Phages, Prophages, Transposable elements, Plasmids” were identified that included two phage tail proteins, two proteins linked to phage replication, and one linked to DNA synthesis. Furthermore, 43 non-assembled sequences were also annotated with the RAST server and compared to the nr database. Among these, six high-identity (>90%) matches with the *Aeromonas* pS68-1 plasmid (CP022182.1) were observed. The AntiSMASH tool identified one homoserine lactone cluster (647,196–667,849 nt), a 20 kb region comprising one core gene, three biosynthetic genes, and three regulatory genes.

### 3.6. Identification and Comparative Analysis of AE59-TE2 Bacteriocin-Related Sequences

The RAST server revealed 101 matches for the virulence, disease, and defense category (Supplementary Table S5). Five of these sequences were annotated into the tolerance to colicin E2 subsystem and nine into the colicin V and bacteriocin production cluster subsystem (Table 2). One predicted sequence (peg.850, 162 aa) exhibited homology to the colicin V production protein (CvpA). Genomic enrichment with the GO FEAT tool revealed four sequences associated with antimicrobial substances: a bacteriocin production protein (CvpA); a Tol-Pal system protein TolQ; a cell envelope integrity protein TolA; and an outer membrane receptor for ferrienterochelin and colicins (Supplementary Table S6). The AE59-TE2 CvpA protein sequence was correlated with a bacteriocin production protein (CvpA) predicted from *A. veronii* (UniProtKB accession number: A0A0T6U8X2) with 98.78% (162/164) coverage and 100% identity. BLAST analysis showed 100% coverage and 100% identity with *Aeromonas* CvpA family protein (accession number: WP_005337086.1), confirming the presence of an important gene related to the production of bacteriocin in strain AE59-TE2. The BLAST analysis against CvpA colicin V production protein (UniProtKB/SwissProt accession number: P08550.1) from *Escherichia coli* str. K-12 substr. MG1655 showed 99% coverage and 64.59% identity.

**Figure 6:**
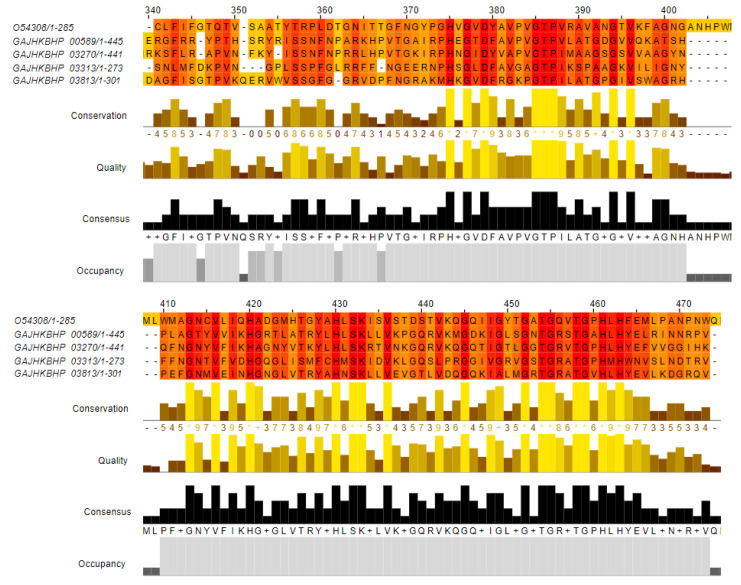
Multiple alignments between Zoocin A protein, a peptidase, from the M23/M37 family from *Streptococcus equi* subsp. *zooepidemicus* (UniProtKB/SwissProt accession number: O54308 and four sequences from the AE59-TE2 genome resulted from BLAST. The positions 340–474 aa show the most conserved region. The Alignment was colored by conservation scheme; the regions with more conserved physicochemical properties are colored in orange (darker color) and the less conserved sites are colored in yellow (lighter color).

BLASTp analyses found four *zooA*-like sequences in the AE59-TE2 genome. The *zooA* gene encodes a Zoocin A protein, a peptidase, from the M23/M37 family from *Streptococcus equi* subsp. *zooepidemicus* (UniProtKB/SwissProt accession number: O54308). One sequence with 50% identity, was annotated as MepM_2 (Murein DD-endopeptidase); another with 46.73% identity, annotated as MepM_1 (Murein DD-endopeptidase); two sequences with 45% and 34.21% identity, were annotated as hypothetical proteins (Table 3). Multiple alignments demonstrated that the most conserved region was between positions 375–500 aa, a region that contains the peptidase M23 domain (Figure 6).

The DoBiscuit database was used to search for more sequences related to antimicrobial activity and found six sequences with more than 60% identity (Table 4).

**Figure 7:**
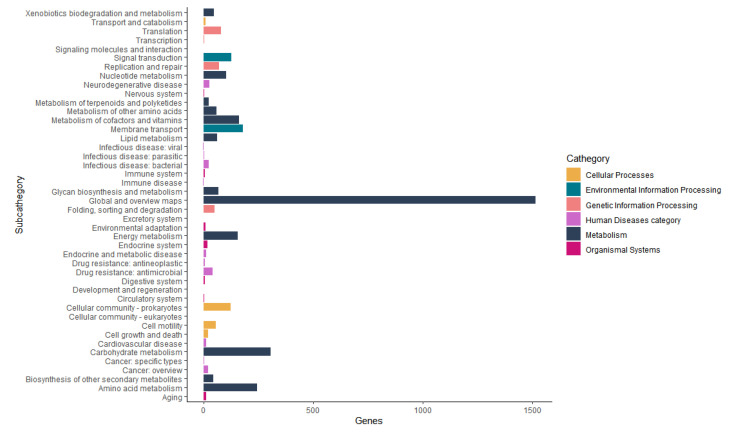
The Functional annotation of orthologous groups with Kaas and KEGG Mapper Reconstruction tools annotated 6 categories of functional groups.

The Functional annotation of orthologous groups with Kaas and KEGG Mapper Reconstruction tools annotated six categories of functional groups (Figure 7). The Metabolism category has 12 subcategories with 2700 genes, amongst these subcategories, the most important for this work are: Metabolism of terpenoids and polyketides with 25 genes and biosynthesis of other secondary metabolites with 44 genes. The biosynthesis of other secondary metabolites subcategories was presented in more detail in Table 5.

Using PROKKA and PATRIC, searches for keywords endopeptidase, endonuclease, polyketide, antibiotic, colicin and microcin unraveled 38 proteins (Table 6). PATRIC identified 129 metabolic pathways within the genome. The most important pathways related to antimicrobial activity were: Biosynthesis of secondary metabolites and Biosynthesis of polyketides and Nonribosomal peptides. In the Biosynthesis of the secondary metabolites category and puromycin Biosynthesis subcategory, a sequence was identified as an *xdhD* gene, a possible hypoxanthine oxidase.

## 4. Discussion

Messi et al., 2003 [22], had previously reported on the potential of *Aeromonas* strains to produce antimicrobial substances. Following their rationale, a screening for antimicrobial activity was performed and confirmed that strains of the *Aeromonas* genus are widely antagonistic, as observed in Figure 1. Thirty-eight of the 57 strains tested demonstrated some type of antagonistic activity towards at least one highly pathogenic bacterial strain. Screening analysis detected a group of seven strains, which can inhibit both gram-positive and gram-negative bacteria, with a different profile from others described in the literature, this is unusual and important for bacteriocin research. A lack of bacteriocin patents suggests they have perhaps been neglected and are an opportunity for novel discoveries. A glance further back in the literature reveals that bacteriocin-producing strains have been described to inhibit *Yersinia ruckeri*, *Listonella anguillarum*, and *Photobacterium damselae* [64]; fish pathogens, such as *Vibrio tubiashii* [65]; and strains associated with food contamination, such as *Staphylococcus* sp. and *Lactobacillus* sp. [21,22].

Strain AE59-TE2 stands out for being able to inhibit 14 of the 16 indicator strains tested. This strain exhibited antagonistic activity towards *K*. *pneumoniae* KPC (*Klebsiella pneumoniae* carbapenemase). KPC-producing bacteria are a group of microorganisms with elevated resistance to various antibiotics, which causes infections that commonly available antibiotics can no longer effectively treat [66]. This result is highlighted, since the majority of bacteriocins come from gram-positive bacteria and are not reported as being antagonistic towards gram-negative pathogenic microorganisms. There is a need for bacteriocins that target gram-negative food-spoilage strains, such as those from the genera *Salmonella* and *Escherichia* [25,26]. These findings shine a light on possible new solutions for medical, pharmaceutical, and food sectors.

In this work, the characterization of the *Aeromonas* AE59-TE2 strain was proposed. Species identification and taxonomy within the *Aeromonas* genus is controversial, even with the contribution of genomic analyses. Based on our data and as described in the literature, *16S rRNA* gene sequences are highly conserved and do not contain enough genetic signal to separate *A. veronii* from *A. allosaccharophila* [12]. Separating these taxons requires more than *16S rRNA* sequence and biochemical tests [67]. DDH analysis and MLSA [45] phylogenetic inference were used with multiple housekeeping genes, including *rpoD*, for taxonomic identification, and the strain was classified as *Aeromonas allosaccharophila* AE59-TE2.

Since *Aeromonas* strains are described as opportunistic pathogens, an equally important factor was assessing the strain’s virulence potential, which could impair its biotechnological applications in the future [11,12]. Aerolysin (*aerA*) [68], toxin A(*rtxA*) [69], layer A (*vapA*) and secretion systems types II (T2SS) (*exeAB* and exeC-N operons), T3SS (*ascV*, *aopP*, *aopH*, *ascC* and *aexT* genes), T4SS (*traB*, *traC*, *traD*, *traE*, *trbJ*, *traA*, *traF*, *traG*, *traH*, *traI*, *traJ* and *traK* genes, with *traA*, *traF*-*traI* as core components) and T6SS (*hcp* (haemolysin), vgrG2 (valine), vgrG (glycine), vgrG1 (ADP-ribosyltransferase activity), *vasH* (transcription regulator) and the *vasK* (unknown function genes) altogether make up for the major virulence factors identified in the *Aeromonas* genus [70,71,72,73].

The virulence factors database (VFDB), from the ABRicate tool, identified the *ascV* and *ascC* genes, which are type III secretion system (T3SS) structural genes, and *aopH*, one of the main effector genes. Vanden Bergh and Frey (2014) [74] demonstrated that, due to several mutations and genetic rearrangements, changes may occur in the type III secretion system. Thus, to affirm its integrity, one must analyze whether the structural genes (*ascV* and *ascC*) are intact and whether the main effector genes (*aopH*, *aexT*, *ati2*, *aopO*, *aopP* and *aopS*) are present [74]. The T3SS is a complex structure used by gram-negative bacteria, which is capable of injecting effector proteins directly into the host cell cytoplasm. Only one effector gene was found in the AE59-TE2 genome. Furthermore, a progressive loss of virulence potential in *A. salmonicida* is observed as constant genetic deletions and additions occur due to horizontal gene transfer with environmental bacteria. This is especially observed in strains grown in laboratories that do not undergo the selective pressures of natural environments [74]. It is worth mentioning that some genes found in the AE59-TE2 genome may not be functional because they are truncated, as has already been described for *Aeromonas* virulence mechanisms [75]. Further analyses found the genes *blaOXA* (a beta-lactamase), *arsD* (an arsenite efflux transporter metallochaperone), *arsC* (glutaredoxin-dependent arsenate reductase), *rsmA* and *adeF* (antibiotic efflux pump), *OXA-726* (beta-lactamase/antibiotic inactivation), and *EF-Tu* (resistance to Pulvomycin). These genes are mostly related to antibiotic resistance. Concerns about virulence with this strain are founded but can be circumvented by using bacteriocins in a purified form. There is increasing interest in the pharmaceutical industry for the use of purified bacteriocins [76].

The antiSMASH tool identified a homoserine lactone cluster in the AE59-TE2 genome. The N-acyl homoserine-lactone (AHL) is a “signal” molecule in gram-negative bacteria and is responsible for the regulation of several biological processes, such as biofilm formation, antibiotic production, and motility [10]. Thus, this is a vital cluster that may be related to the antimicrobial activity observed in our analyses.

RAST server annotation uncovered a CvpA protein in the AE59-TE2 genome. This protein is required for colicin V production and was originally identified in plasmid pColV-K30 from *Escherichia coli*. Nonetheless, this is not the structural gene for the colicin V bacteriocin [77]. The *cvpA* gene is chromosomal and is required for colicin V production and secretion 77]. It encodes an inner membrane protein that is involved in the colicin V export machinery [77]. The colicin V structural *cvaC* gene and the *cvaA* and *cvaB* genes are required for toxin processing and export. The protein that confers immunity on the host cell is encoded by the *cvi* gene [78]. Gene clusters similar to known bacteriocins have been described in other *Aeromonas* genomes [79], and the receptor for ferrienterochelin and colicins was identified in *A. salmonicida* subsp. *pectinolytica* 34melT genome [80]. However, no correlation between the presence of these clusters and bacteriocin activity has been reported until now. BLAST analysis between a CvpA protein identified in the AE59-TE2 genome and a CvpA colicin V production protein from *Escherichia coli* str. K-12 substr. MG1655 revealed a 64.59% identity. This result suggests that the gene could be associated with the production and secretion of colicin V peptide, *cvaC* gene, or a similar structural peptide gene [78]. However, no significant homology to the *E. coli cvaC* gene was found.

Blast analyses identified four sequences with similarities to the *zooA* gene. This gene encodes a Zn-metalloprotease called zoocin A, belonging to the M23/M37 family, and isolated initially from *Streptococcus equi* subsp. *zooepidemicus*. This protein functions as an enzybiotic that is active against gram-positive bacteria, cleaving peptides from their cell wall [81]. The AE59-TE2 strain was able to inhibit the growth of gram-positive bacteria, namely *Enterococcus* sp. and *Staphylococcus* sp., which is not a common feature for gram-negative bacteriocin-producing strains. These data suggest that the AE59-TE2 strain might use different mechanisms to inhibit gram-negative and gram-positive bacteria. Multiple alignments between the four sequences found in the AE59-TE2 genome and other zooA sequences from the UniProt database demonstrated a highly conserved region for a peptidase M23 domain, suggesting that they may be new sequences related to the production of bacteriocins similar to Zoocin A.

The BLAST against DoBiscuit Database resulted in six sequences with more than 60% identity with sequences related to antibiotics. These sequences were annotated in the PROKKA software as RpoC, InfA (translation initiation factor IF-1), ThiC (phosphomethylpyrimidine synthase), FadH (2,4-dienoyl-CoA reductase), and MetK (S-adenosylmethionine synthase). Proteins RpoC and InfA could be related with resistance to Ansamycin and Rubradirin, respectively. The ThiC protein is associated with thiamine biosynthesis. The FadH protein is a NADPH-dependent 2,4-dienoyl-CoA reductase, and MetK protein catalyzes the formation of S-adenosylmethionine (AdoMet) from methionine and ATP and is associated with tylosin production [82]. Tylosin is a macrolide antibiotic that is used as a feed additive in veterinary medicine.

KEGG analysis provided a general characterization of the genome and highlighted several important pathways to be studied. These results were further explored by investigating and comparing sequences with genes of known important antimicrobial compounds. For instance, the peptidase family C39 contains bacteriocin processing endopeptidases. In this genome, the *mepM* gene was identified and is related to peptidoglycan synthesis [14]. Also identified was the *nlpC* gene, which is related to cell wall remodeling, cell separation during division, and cleaving non-canonical peptide bonds [83]. Zoocin A is a D-alanyl-L-alanyl endopeptidase, which hydrolyses cross bridges in the peptidoglycan structure of susceptible *streptococci* [84]. The PROKKA software identified two sequences as D-alanyl-D-alanine endopeptidases (GAJHKBHP_00392 and GAJHKBHP_03927), corroborating with previous results of Zoocin A sequences. One protein containing an HNH endonuclease domain (Peg.1792) was annotated by the PATRIC server. HNH-type endonucleases are known as Nuclease Bacteriocins (NB) [85]. Polyketides (PKS) were also pursued due to their antimicrobial activity, as described in the literature. Kegg analysis identified the rfb operon, which comprises four genes (*rfbABCD*) and is involved in dTDP-rhamnose biosynthesis. Genes *rfbAB* transform D-glucose-1-phosphate into dTDP-4-oxo-6-deoxy-D-glucose, an essential substance in polyketide sugar unit biosynthesis. This substance is further processed by genes *rfbCD*, resulting in dTDP-l-rhamnose. This latter substance can be involved in the biosynthesis of enediyne antibiotics and streptomycin. Streptomycin, for instance, is an aminoglycoside that possesses antimicrobial activity towards many bacteria, such as *Bacillus subtilis*, *E. coli*, certain strains of *Salmonella*, *B. mycoides*, *B. cereus*, and *P. aeruginosa* [86]. Several enzyme complexes can be produced by the secondary metabolism of bacteria. Type I PKSs, known as modular/iterative, are multicatalytic enzymes, which give rise to known natural products, such as macrolides (erythromycin) and polyenes (nystatin). On the other hand, type II aromatic PKSs are mono and bifunctional enzymes that interact during the synthesis of polycyclic aromatic compounds, such as tetracycline or doxorubicin [87]. Polyketide synthase modules and other related proteins were annotated in the PATRIC server (peg.1909). Antibiotic biosynthesis monooxygenase (ABM) is a protein superfamily that is involved in the production of several antibiotics, playing an important role in the biosynthesis of aromatic polyketides. ABM leads to a significant increase in antibiotic production [88]. The PATRIC server identified an antibiotic biosynthesis monooxygenase (peg.705), demonstrating another important sequence related to antimicrobial activity and how rich is the genome. The PATRIC server also demonstrated several important pathways related to antimicrobial activity to be further explored in the future.

These genomic analyses of an *A. allosaccharophila* strain fill in a knowledge gap for this species, which has not been studied in such detail before. Furthermore, the *A. allosaccharophila* AE59-TE2 genome has similarities with the enzybiotic zoocin A endopeptidase sequences from streptococci bacteria. AE59-TE2 possesses a broad spectrum of inhibitory activity, targeting gram-positive and gram-negative multidrug resistant pathogens. Genomic analyses revealed important sequences associated with antimicrobial activity. Further analyses are required to better elucidate this antimicrobial substance, since it holds promising biotechnological use for the health and food sectors.

## Figures and Tables

**Table 1:** AE59-TE2 genome assembly statistics. These values represent the main output of each assembly step, starting with the de novo assembly carried out with SPAdes software and followed by two runs of scaffolding conducted with the Medusa online server and *A. veronii* complete genomes as references. The result generated after the second scaffolding run represents the final AE59-TE2 assembly. A flowchart of assembly steps is shown in Supplementary Figure S2.

Statistics	De Novo Assembly	Scaffolding 1	Scaffolding 2
Number of sequences	109	62	51
Largest contig (bp)	473,819	3,152,962	4,498,261
Average length (bp)	41,433.55	72,878.34	88,608.96
Total length (bp)	4,516,257	4,518,457	4,519,057
% GC	58.65	58.63	58.62
N50	263,685	3,152,962	4,498,261

**Table 2:** Bacteriocin-related genes of AE59-TE2 identified by RAST functional annotation tool.

Subsystem	Putative Function	Gene ID
Colicin V and Bacteriocin Production Cluster	DedA protein	peg.679
		peg.2991
		peg.1442
	Amidophosphoribosyltransferase (EC 2.4.2.14)	peg.851
	Colicin V production protein	peg.850
	DedD protein	peg.934
	Folylpolyglutamate synthase (EC 6.3.2.17); Dihydrofolate synthase (EC 6.3.2.12)	peg.933
	Acetyl-coenzyme A carboxyl transferase beta chain (EC 6.4.1.2)	peg.932
	tRNA pseudouridine synthase A (EC 4.2.1.70)	peg.931
Tolerance to colicin E2	Conserved uncharacterized protein CreA	peg.2597
		peg.2682
	Two-component response regulator CreB	peg.2016
	Two-component response regulator CreC	peg.2017
	Inner membrane protein CreD	peg.2020

**Table 3:** BLAST result between AE59-TE2 annotation and bacteriocins from UniProt Database.

Result of BLASTp AE59-TE2 (Amino Acid by Prokka Annotation) X Bacteriocins DB											
Sequence from AE59 (Query)	% Identity	Subject Acc.Ver	Alignment Length	Mismatches	Gap Opens	q. Start	q. End	s. Start	s. End	E-Value	Bit Score
GAJHKBHP_00589	46.73%	Zoocin A (Uniprot Acc. Number: O54308)	92	42	2	319	403	22	113	2.08 × 10^−19^	79.0
GAJHKBHP_03270	50.00%		94	38	2	301	386	45	137	2.81 × 10^−20^	82.4
GAJHKBHP_03313	34.21%		114	58	3	171	268	45	157	8.07 × 10^−17^	70.5
GAJHKBHP_03813	45.0%		100	47	3	192	284	39	137	6.67 × 10^−17^	71.2

**Table 4:** BLAST result between AE59-TE2 annotation and sequences from the DoBiscuit Database.

Result of BLASTp AE59 (Prokka Annotation) X DoBiscuit Database						
Sequence from AE59 (Query)	% Identity	Subject	Classified	Relative	PROKKA Gene	PROKKA Product
GAJHKBHP_00274	73.33%	Rifam_00640	PKS TypeI modular	Ansamycin	* rpoC *	DNA-directed RNA polymerase subunit beta
GAJHKBHP_02296	63.01%	Rubra_00090	PKS TypeI modular	Rubradirin	*infA*	Translation initiation factor IF-1
GAJHKBHP_00363	62.65%	A4092_00490	NRPS/PKS TypeIII	Glycopeptide(teicoplanin-type)	* thiC *	Phosphomethylpyrimidine synthase
GAJHKBHP_01234	61.41%	Salino_00470	PKS TypeI modular	Salinomycin/Polyether	* fadH *	2% 2C4-dienoyl-CoA reductase
GAJHKBHP_02296	61.19%	Rubra_00040	PKS TypeI modular	Rubradirin/Ansamycin	*infA*	Translation initiation factor IF-1
GAJHKBHP_03276	60.16%	Polk_00010	PKS TypeI iterative	Tetracyclic quinone glycoside	*metK*	S-adenosylmethionine synthase
			PKS TypeII	Polyketomycin		

**Table 5:** List of KEGG Biosynthesis of secondary metabolites Reference pathway.

Biosynthesis of Secondary Metabolites	Pathway Modules
Macrolide biosynthesis	M00773 Tylosin biosynthesis
	M00934 Mycinamicin biosynthesis
	M00774 Erythromycin biosynthesis
	M00775 Oleandomycin biosynthesis
	M00776 Pikromycin/methymycin biosynthesis
	M00777 Avermectin biosynthesis
Type II polyketide biosynthesis	M00778 Type II polyketide backbone biosynthesis
	M00779 Dihydrokalafungin biosynthesis
	M00780 Tetracycline/oxytetracycline biosynthesis
	M00823 Chlortetracycline biosynthesis
	M00781 Nogalavinone/aklavinone biosynthesis
	M00782 Mithramycin biosynthesis
	M00783 Tetracenomycin C/8-demethyltetracenomycin C biosynthesis
	M00784 Elloramycin biosynthesis
Biosynthesis of beta-lactams	M00672 Penicillin biosynthesis
	M00673 Cephamycin C biosynthesis
	M00675 Carbapenem-3-carboxylate biosynthesis
	M00736 Nocardicin A biosynthesis
	M00674 Clavaminate biosynthesis
Biosynthesis of other antibiotics	M00877 Kanosamine biosynthesis glucose 6-phosphate => kanosamine
	M00889 Puromycin biosynthesis
	M00815 Validamycin A biosynthesis
	M00904 Dapdiamides biosynthesis
	M00785 Cycloserine biosynthesis
	M00787 Bacilysin biosynthesis
	M00848 Aurachin biosynthesis
	M00788 Terpentecin biosynthesis
	M00819 Pentalenolactone biosynthesis
	M00903 Fosfomycin biosynthesis
	M00890 Roseoflavin biosynthesis

**Table 6:** Result keyword searches using PATRIC and PROKKA annotation. The keywords that were searched: “endopeptidase, endonuclease, polyketide (PKS), antibiotic, colicin, microcin, zoocin, bacteriocin and streptomycin”.

Results of Searches for Keywords: “Endopeptidase, Endonuclease, Polyketide, Antibiotic, Colicin and Microcin”			
Keyword	Annotation Tool	Sequence ID	Product
Endonuclease	PATRIC	peg.3314	Endonuclease I precursor/deoxyribonuclease I activity
	PATRIC	peg.2963	Endonuclease IV/deoxyribonuclease IV (phage-T4-induced) activity
	PATRIC	peg.2394	Predicted ATP-dependent endonuclease of the OLD family, YbjD subgroup
	PATRIC	peg.2112	DNA/RNA endonuclease G
	PATRIC	peg.1965	Endonuclease III/DNA-(apurinic or apyrimidinic site) lyase activity
	PATRIC	peg.1792	Protein containing HNH endonuclease domain
	PATRIC	peg.1779	Esterase ybfF
	PATRIC	peg.1125	Flap endonuclease Xni
	PATRIC	peg.512	DNA mismatch repair endonuclease MutH
	PROKKA	GAJHKBHP_01017	putative DNA endonuclease SmrA
	PROKKA	GAJHKBHP_01117	Flap endonuclease Xni-YgdG
	PROKKA	GAJHKBHP_01958	Endonuclease III-Nth
	PROKKA	GAJHKBHP_02669	Endonuclease MutS2
	PROKKA	GAJHKBHP_02932	Endonuclease 4-Nfo
Endopeptidase	PATRIC	peg.384	Murein-DD-endopeptidase
	PATRIC	peg.577	Murein DD-endopeptidase MepM
	PATRIC	peg.2124	Probable endopeptidase NlpC
	PATRIC	peg.2264	Penicillin-insensitive murein endopeptidase
	PATRIC	peg.3967	Murein-DD-endopeptidase (EC 3.4.99.-)
	PROKKA	GAJHKBHP_00392	D-alanyl-D-alanine endopeptidase-PbpG_1
	PROKKA	GAJHKBHP_00589	Murein DD-endopeptidase MepM
	PROKKA	GAJHKBHP_02126	Oligoendopeptidase F% 2C plasmid-PepF1
	PROKKA	GAJHKBHP_02243	Penicillin-insensitive murein endopeptidase-MepA_1
	PROKKA	GAJHKBHP_03259	Neutral endopeptidase-PepO
	PROKKA	GAJHKBHP_03270	Murein DD-endopeptidase MepM
	PROKKA	GAJHKBHP_03927	D-alanyl-D-alanine endopeptidase-PbpG_2
Colicin	PATRIC	peg.1689	Colicin I receptor precursor
	PATRIC	peg.2329	Colicin V production protein
	PROKKA	GAJHKBHP_00287	Colicin I receptor-CirA_1
	PROKKA	GAJHKBHP_01798	Colicin I receptor-CirA_2
	PROKKA	GAJHKBHP_01806	Colicin I receptor-CirA_3
	PROKKA	GAJHKBHP_02310	Colicin V production protein-CvpA
Antibiotic	PATRIC	peg.705	Antibiotic biosynthesis monooxygenase
	PROKKA	GAJHKBHP_03466	Phenazine antibiotic resistance protein EhpR
Microcin	PATRIC	peg.2417	Microcin C7 immunity MccF-like protein
	PROKKA	GAJHKBHP_02397	Microcin C7 self-immunity protein MccF
Polyketide	PATRIC	peg.1909	Polyketide synthase modules and related proteins
	PATRIC	peg.2168	Polyketide synthase modules and related proteins

## Data Availability

The Whole Genome Shotgun (WGS) project of *Aeromonas allosaccharohila* strain AE59-TE2 has been deposited at GenBank under the accession CP090911. The AE59-TE2 BioSample accession number is SAMN08436981 and the Bioproject accession number is PRJNA432149.

## Supplementary Materials

The following supporting information can be downloaded at: https://www.mdpi.com/article/10.3390/life12101492/s1, Supplementary Figure S1: Antibacterial activity of strain AE59-TE2 against *Escherichia coli*. Nutrient agar plate containing two producers of antimicrobial substances (AE59-TE2 and AE38.XC4), showing an inhibition halo against the *E. coli* indicator bacteria. The method used was the agar diffusion assay (Tagg and McGiven, 1971); Supplementary Figure S2: Flowchart depicting the assembly steps; Supplementary Table S1: *Aeromonas* type strain obtained from EZBio Cloud and used for MLSA phylogenetic tree construction, in silico DDH and ANI. Table.; Table S2: Statistics for raw and filtered reads, Table.; Supplementary Table S3: Results of in silico DDH analysis carried out with GGDC tool; Supplementary Table S4: Result of search for virulence genes with virulence factors database (VFDB) from ABRicate tool; Supplementary Table S5: AE59-TE2 genes annotated in the “Virulence, Disease and Defense” RAST category; Supplementary Table S6: Result of putative bacteriocins sequences identified by BOA tool and compared with RAST, GoFeat tool and BACTIBASE tool result.
