# Supplementary figures and images for: Aeromonas allosaccharophila Strain AE59-TE2 Is Highly Antagonistic towards Multidrug-Resistant Human Pathogens, What Does Its Genome Tell Us?
Source: Life (Basel). 2022 Sep 26;12(10):1492. doi: 10.3390/life12101492 (PMC9605075; doi:10.3390/life12101492)

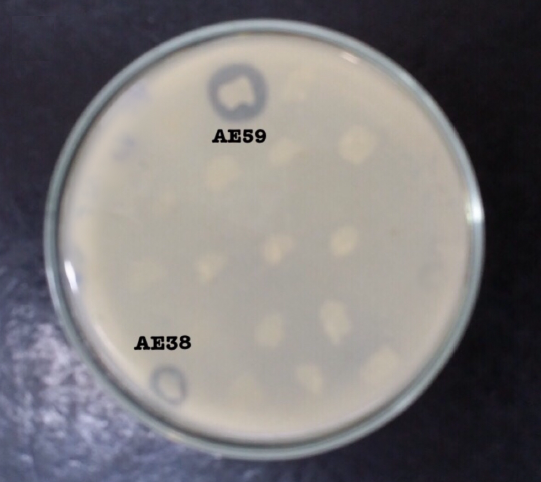

Supplement: Supplementary file 1 [file life-12-01492-s001.zip › life-1917417-supplementary/Figure S1.png]

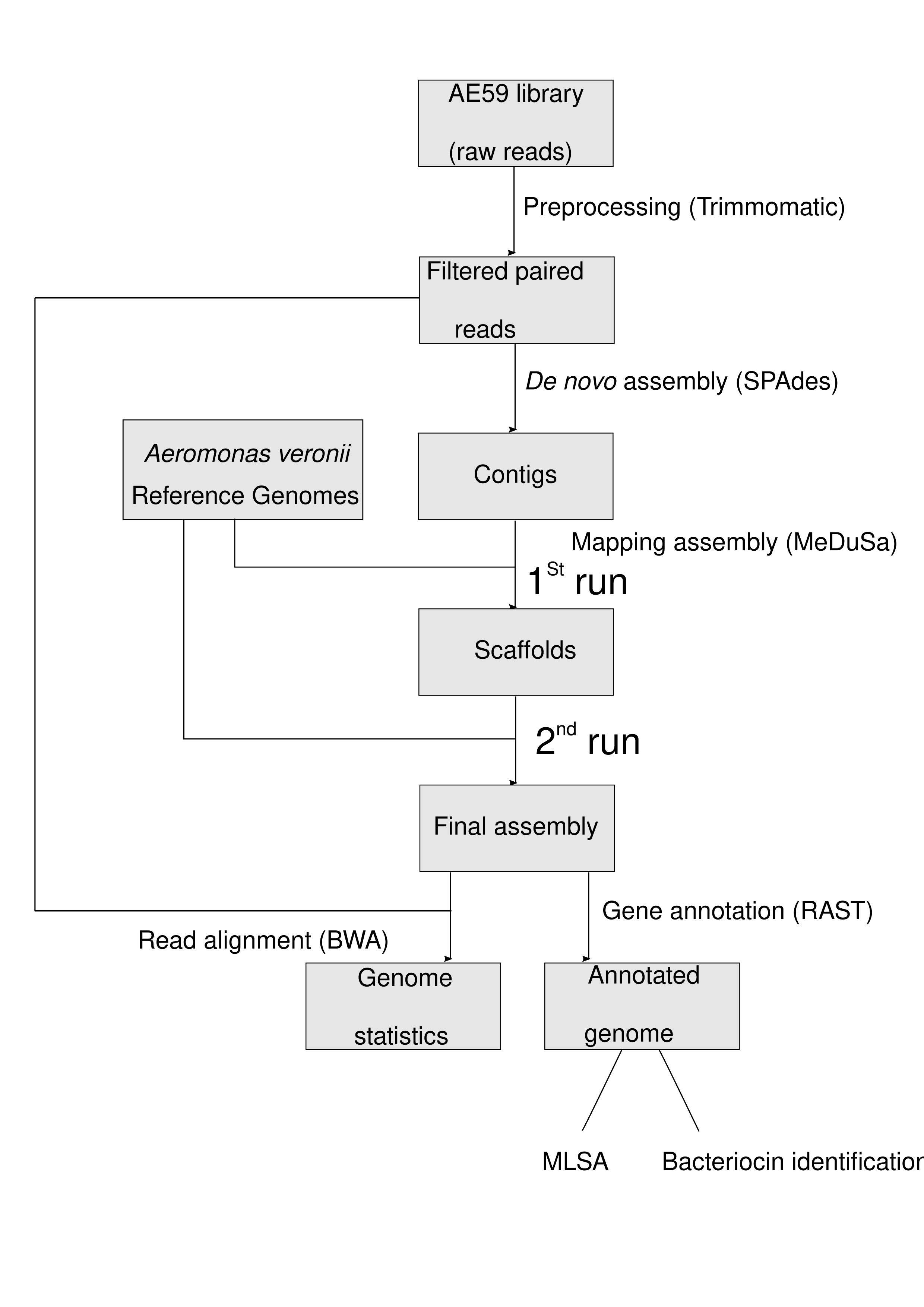

Supplement: Supplementary file 1 [file life-12-01492-s001.zip › life-1917417-supplementary/Figure S2.png]
